# Assessment of Tau Tangles and Amyloid-β Plaques Among Super Agers Using PET Imaging

**DOI:** 10.1001/jamanetworkopen.2020.28337

**Published:** 2020-12-11

**Authors:** Merle C. Hoenig, Niclas Willscheid, Gérard N. Bischof, Thilo van Eimeren, Alexander Drzezga

**Affiliations:** 1 Research Center Juelich, Institute for Neuroscience and Medicine II, Molecular Organization of the Brain, Juelich, Germany; 2 Department of Nuclear Medicine, Faculty of Medicine and University Hospital Cologne, University of Cologne, Cologne, Germany; 3 Department of Neurology, Faculty of Medicine and University Hospital Cologne, University of Cologne, Cologne, Germany; 4 German Center for Neurodegenerative Diseases, Bonn, Germany

## Abstract

This cross-sectional study examines positron emission tomography (PET) imaging to investigate the burden of tau tangles and amyloid β plaques in super agers, normal agers, and patients with mild cognitive impairment vs younger amyloid-negative controls.

## Introduction

Little is known about the presence and extent of amyloid-β plaques and tau tangles in individuals who preserve exceptional cognitive function despite advanced age,^1^ also known as super agers. Although lower expression of these hallmarks of neurodegeneration may be expected in this group,^2,3,4^ in vivo evidence of tau tangles in particular is still lacking. Using positron emission tomography (PET) imaging data, we therefore studied the tau tangle and amyloid-β plaque burdens in a super ager (SA) group, normal ager (NA) group, and a patient group with mild cognitive impairment (MCI). Because tau tangles are closely associated with cognitive decline, we hypothesized that the SA group would have fewer tau tangles than the NA and MCI groups.

## Methods

In this cross-sectional study, we included data (retrieved in June 2019) of 3 age- and education-matched patient groups of 25 SAs, 25 NAs, and 25 patients with MCI, all aged 80 years or older (Table) from the Alzheimer’s Disease Neuroimaging Initiative (ADNI) database. A group of younger amyloid-negative controls (YC group) served as the reference. Ethical approval was obtained by the ADNI investigators at each participating site. All participants provided written informed consent.

**Table.** Demographic Characteristics of the Studied Groups

Characteristics	SA	NA	MCI	YC	*P* value^a^		
					SA vs NA	SA vs MCI	NA vs MCI
Participants, No.	25	25	25	19	.32	.19	.02
Men	14	10	19	3	N/A	N/A	N/A
Women	11	15	6	16	N/A	N/A	N/A
Age at tau scan, mean (SD), y^b^	85.21 (3.51)	84.52 (3.49)	84.77 (3.93)	63.60 (2.76)	.17	.10	.89
Years of education, mean (SD)	17.40 (2.58)	16.40 (2.63)	16.16 (2.73)	16.31 (2.77)	.41	.58	.71
ApoE4 carrier (yes/no)	5/20	5/20	9/16	2/14^c^	.94	.24	.24
Memory score at tau scan, mean (SD)	1.34 (0.37)	.61 (0.29)	−.54 (0.44)	N/A	<.001	<.001	<.001

Abbreviations: ApoE4, apolipoprotein E4; MCI, mild cognitive impairment; NA, normal agers; N/A, not applicable; SA, super agers; YC, younger control group.

^a^ *P* values are depicted for the group comparisons.

^b^ Age at tau positron emission tomography scan acquisition.

^c^ For the 3 individuals in this group no information on ApoE4 status was available.

We categorized the SA, NA, and MCI groups according to the ADNI memory score from the ADNI neuropsychological test battery.^5^ We focused on at least 4 ADNI memory score measurements within a 4-year period leading back from the [^18^F]AV-1451 PET acquisition. Individuals with a mean ADNI memory *z* score greater than 1.25 were defined as the SA group, those with a mean *z *score between 0.5 and 1.25 during the study period were defined as the NA group, and those with a mean *z *score of less than 0 were defined as the MCI group.

Regional group differences in tau tangles and amyloid-β plaques were compared between the YC group and the other 3 groups using normalized and intensity-standardized (reference: cerebellum) [^18^F]AV-1451 (tau) and [^18^F]AV-45 (amyloid) PET scans in a voxelwise (*P* < .0001, uncorrected) and a region-of-interest (ROI) approach, including sex as covariate. The ROI approach included 5 meta-ROIs (entorhinal cortex, inferior temporal, middle occipital, precuneus, and orbitofrontal gyrus) and was corrected for multiple comparisons (Bejamini Hochberg correction).^6^ Statistical testing was 2-sided with *P* < .05 considered statistically significant and analysis of covariance corrected for sex. Final statistical analysis was performed using SPSS version 25 (IBM Corp) in September 2020.

## Results

There were 94 participants, including 48 women (51.06%). The mean (SD) age was 85.21 (3.51) years for the SA group, 84.52 (3.49) years for the NA group, 84.77 (3.93) years for the MCI group, and 63.60 (2.76) years for the YC group.

The results of the voxelwise analysis (Figure, A) yielded no differences in tau tangles and amyloid-β plaques when comparing the SA group with the YC group. In contrast, the NA group presented with higher tau burden in medial temporal regions but no differences in amyloid burden compared with YC group. The MCI group demonstrated both elevated amyloid and tau burden. Significant differences of the ROI analysis surviving multiple comparison correction (Figure, B) accorded with the results of the voxelwise analysis. The NA group had more tau tangles in entorhinal (*F*_1,40_ = 19.808; *P* < .001), inferior temporal (*F*_1,40_ = 22.461; *P* < .001), and orbitofrontal (*F*_1,40_ = 5.698; *P* = .02) regions, and the MCI group presented overall greater pathogenic burden (*P* = .01) except tau tangles in the orbitofrontal region (*F*_1,40_ = 2.128; *P* = .15) and amyloid plaques in the entorhinal region (*F*_1,40_ = 3.484; *P* = .07) compared with the YC group. Direct comparison of NAs vs SAs yielded significantly higher inferior temporal (*F*_1,45_ = 7.45; *P* = .009) and precuneal (*F*_1,45_ = 7.74; *P* = .008) tau tangles in the NA group.

**Figure.**
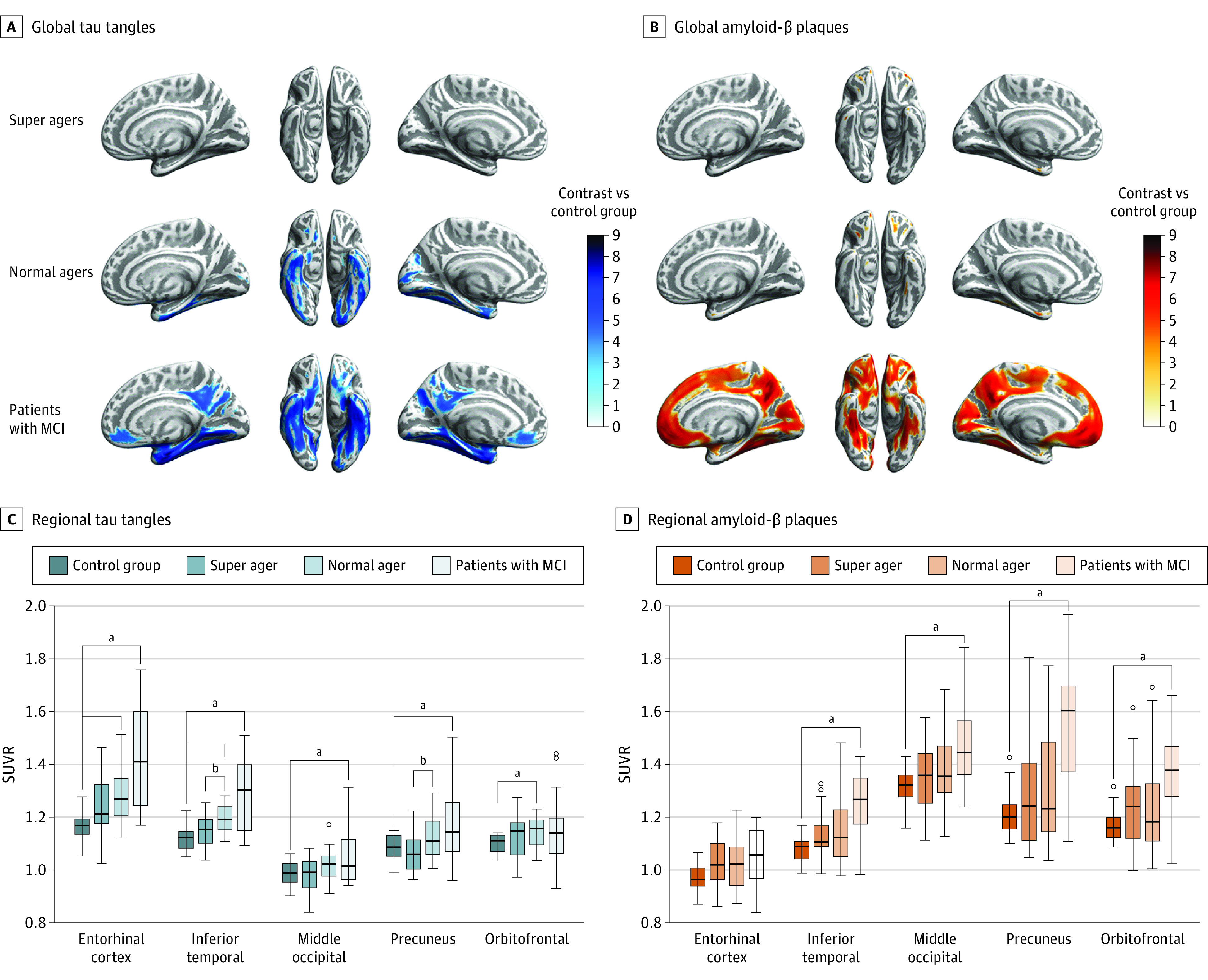
Global and Regional Tau Tangles and Amyloid β Plaques for the Super Ager, Normal Ager, and Mild Cognitive Impairment (MCI) Groups A and B, All brain projections represent the contrast of the respective group against the younger healthy cognitively normal group (*P* < .0001, uncorrected). C and D, Box plots show significant differences between regional standard uptake value ratios (SUVRs) of the 4 groups. Lines within boxes denote medians, tops and bottoms of boxes denote 75th and 25th percentiles, error bars denote 95% CIs, and circles denote outliers. Benjamini Hochberg corrected *P* value threshold was *P* ≤ .022 for tau burden and *P* ≤ .007 for amyloid burden. ^a^Comparison of respective group vs younger healthy control group. ^b^Comparison of super agers vs normal agers.

## Discussion

The in vivo findings of this cross-sectional study suggest that the phenomenon of super aging may be associated with higher brain resistance against the buildup of both tau tangles and amyloid-β plaques, which could prevent neurodegeneration, as previously hypothesized.^1,3^ Normal aging, in contrast, appears to be associated with tau tangles but not amyloid plaques, pointing to a role of isolated tau accumulation in age-related cognitive decline, whereas synergistic effects of both proteinopathies seem to accelerate the unsuccessful aging process as seen in MCI.

A limitation of this study was the small sample size. Despite the small sample size and the cross-sectional design, this study may stimulate future longitudinal assessments in larger, less selective cohorts also examining the role of lifestyle and molecular pathways, to decipher causal factors associated with successful aging. Overall, the characterization of individuals who remain resistant to these aging-associated proteinopathies, may inspire novel concepts for cognitive preservation in older age and therapy of neurodegeneration.

## References

[1] Rogalski E, Gefen T, Mao Q, Cognitive trajectories and spectrum of neuropathology in superagers: the first 10 cases. Hippocampus. 2019;29(5):458-467. doi:10.1002/hipo.22828 29341318 PMC6050141

[2] Gefen T, Peterson M, Papastefan ST, Morphometric and histologic substrates of cingulate integrity in elders with exceptional memory capacity. J Neurosci. 2015;35(4):1781-1791. doi:10.1523/JNEUROSCI.2998-14.2015 25632151 PMC4308613

[3] Dang C, Yassi N, Harrington KD, ; AIBL Research Group Rates of age- and amyloid β-associated cortical atrophy in older adults with superior memory performance. Alzheimers Dement (Amst). 2019;11(1):566-575. doi:10.1016/j.dadm.2019.05.005 31909172 PMC6939054

[4] Dekhtyar M, Papp KV, Buckley R, Neuroimaging markers associated with maintenance of optimal memory performance in late-life. Neuropsychologia. 2017;100:164-170. doi:10.1016/j.neuropsychologia.2017.04.037 28472627 PMC5522601

[5] Crane PK, Carle A, Gibbons LE, ; Alzheimer’s Disease Neuroimaging Initiative Development and assessment of a composite score for memory in the Alzheimer’s Disease Neuroimaging Initiative (ADNI). Brain Imaging Behav. 2012;6(4):502-516. doi:10.1007/s11682-012-9186-z 22782295 PMC3806057

[6] Benjamini Y, Hochberg Y Controlling the false discovery rate: a practical and powerful approach to multiple testing. J Royal Stat Soc. 1995;57(1):289-300.

